# Proliferation zones in the axolotl brain and regeneration of the telencephalon

**DOI:** 10.1186/1749-8104-8-1

**Published:** 2013-01-17

**Authors:** Malcolm Maden, Laurie A Manwell, Brandi K Ormerod

**Affiliations:** 1 Department of Biology & UF Genetics Institute, University of Florida, PO Box 118525, Gainesville, FL, 32611, USA; 2 J. Crayton Pruitt Family Department of Biomedical Engineering, Department of Neuroscience and McKnight Brain Institute, University of Florida, Gainesville, FL, USA; 3 Department of Psychology, Sir Wilfrid Laurier University, Waterloo, ON, Canada

**Keywords:** Axolotl, Brain regeneration, DCX, GFAP, NeuN, Neural precursor cells, Telencephalon, Ventricular zone

## Abstract

**Background:**

Although the brains of lower vertebrates are known to exhibit somewhat limited regeneration after incisional or stab wounds, the Urodele brain exhibits extensive regeneration after massive tissue removal. Discovering whether and how neural progenitor cells that reside in the ventricular zones of Urodeles proliferate to mediate tissue repair in response to injury may produce novel leads for regenerative strategies. Here we show that endogenous neural progenitor cells resident to the ventricular zone of Urodeles spontaneously proliferate, producing progeny that migrate throughout the telencephalon before terminally differentiating into neurons. These progenitor cells appear to be responsible for telencephalon regeneration after tissue removal and their activity may be up-regulated by injury through an olfactory cue.

**Results:**

There is extensive proliferation of endogenous neural progenitor cells throughout the ventricular zone of the adult axolotl brain. The highest levels are observed in the telencephalon, especially the dorsolateral aspect, and cerebellum. Lower levels are observed in the mesencephalon and rhombencephalon. New cells produced in the ventricular zone migrate laterally, dorsally and ventrally into the surrounding neuronal layer. After migrating from the ventricular zone, the new cells primarily express markers of neuronal differentiative fates. Large-scale telencephalic tissue removal stimulates progenitor cell proliferation in the ventricular zone of the damaged region, followed by proliferation in the tissue that surrounds the healing edges of the wound until the telencephalon has completed regeneration. The proliferative stimulus appears to reside in the olfactory system, because telencephalic regeneration does not occur in the brains of olfactory bulbectomized animals in which the damaged neural tissue simply heals over.

**Conclusion:**

There is a continual generation of neuronal cells from neural progenitor cells located within the ventricular zone of the axolotl brain. Variable rates of proliferation were detected across brain regions. These neural progenitor cells appear to mediate telencephalic tissue regeneration through an injury-induced olfactory cue. Identification of this cue is our future goal.

## Background

During vertebrate central nervous system (CNS) development, cells of the pseudostratified epithelium undergo cytokinesis at the ventricular surface. One daughter cell migrates peripherally along radial glial cells, whose processes extend from the ventricular to the pial surface, to differentiate in the mantle layer while the other daughter cell is retained to continue proliferating at the ventricular surface, eventually forming the ventricular zone (VZ). In mammals, this VZ separates into two layers: the ependymal cells forming a single layer in contact with the lumen, and the subventricular zone.

Although cytokinesis persists throughout the adult mammalian neuraxis [1,2], neuronal differentiation occurs only in two restricted regions of the telencephalon. Neural progenitor cells (NPCs) resident in the subventricular zone chain migrate as neuroblasts over long distances along the rostral migratory stream to the olfactory bulbs, where they terminally differentiate into GABAergic granule neurons and dopaminergic periglomerular neurons [3]. NPCs resident in the hippocampal subgranular zone differentiate into glutamatergic granule neurons of the granule cell layer and a few hilar astrocytes [4]. Although NPCs capable of generating neurons in culture can be harvested from the entire neuraxis of living (throughout life) and cadaveric mammalian CNS tissue [4-8], they only spontaneously generate significant numbers of neurons *in vivo* in the hippocampus and olfactory bulbs. In lower vertebrates such as reptiles, amphibians and fish, VZ NPCs are thought to proliferate throughout adulthood [9-12]. A comparative approach that examines the distribution of proliferating NPCs and the differentiation and migration of their progeny in lower vertebrates may reveal mechanisms that could be harnessed to stimulate neuronal regeneration in the adult mammalian CNS.

Precise localization labeling experiments in zebrafish and other teleosts [13] have revealed proliferating NPCs in VZs throughout the major subdivisions of the adult brain with the exception of the hypothalamus and cerebellum, where proliferative zones are located deeper in the parenchyma [14,15]. Proliferation rates are faster in ventral versus dorsal regions of the teleost telencephalon, where cells migrate centrifugally away from the VZ as they differentiate into neurons that integrate into neuronal circuits [15-19]. Ventral telencephalic neuroblasts migrate through a rostral migratory stream that resembles the mammalian rostral migratory stream, before terminally differentiating into neurons in the olfactory bulbs [16,20]. Although teleost brain regeneration is far less studied than optic nerve or retinal regeneration, a stab wound can up-regulate VZ NPC proliferation, which is followed by the migration of new cells into damaged telencephalic regions [21-25]. In the knifefish, an incisional cerebellar wound stimulates proliferation at the site of injury that is followed by the migration of new cells along radial glial fibers into the wound [13].

In reptiles, spontaneous but variable rate NPC proliferation has been noted in the VZs of the telencephalon and cerebellum [26,27]. In a manner similar to that noted in teleosts, neuroblasts (but not glioblasts) migrate centrifugally away from the VZ and are thought to migrate through a rostral migratory stream into the olfactory bulbs before terminally differentiating into neurons. In reptiles, incisional wounds can stimulate the proliferation of VZ NPCs that appear to induce relatively slow and incomplete wound repair [28]. In the lizard, with more drastic dorsal telencephalic segment removal some tissue regeneration is noted, but with limited cell layering, even after 260 days [29].

The amphibian telencephalon contains a dorsal and thicker ventral matrix (ventricular) zone [9,30] that exhibits higher proliferative and regenerative capacity than the teleost and reptile telencephalon VZ. In the adult newt, NPCs proliferate in the telencephalon VZ anterior to the quiescent mesencephalon, hindbrain and cerebellum regions [31,32]. Removal of 70% of the optic tectum in these animals induces rapid tissue regeneration followed by more prolonged retinotectal projection regeneration [33]. In addition, their mesencephalic dopaminergic neurons are completely regenerated after 6-hydroxydopamine-induced ablation stimulates proliferation among normally quiescent mesencephalic ependymoglia [31,32]. The remarkable regenerative ability of the axolotl telencephalon has been characterized most extensively by Kirsche [9,34-36] and others [37,38]. They found that telencephalic segments can regenerate providing the olfactory nerves are intact, and concluded that the stimulatory cue to regeneration provided by the olfactory system is the cellular contribution provided by olfactory neuroblasts. Although the olfactory epithelium replaces lost neurons throughout life [39], and transplanted olfactory epithelial ensheathing cells can minimize CNS lesions [40], there is little evidence in teleosts, reptiles or mammals that olfactory epithelial cells migrate into brain parenchyma. Rather, telencephalon VZ NPCs produce neuroblasts that migrate toward the olfactory bulbs in these species. Therefore, the olfactory nerve or bulb more likely provides an injury-induced cue that up-regulates spontaneous VZ NPC proliferation to induce plasticity and regeneration in the Urodele brain.

Here, we conduct a detailed study of the distribution of NPCs that divide spontaneously and in response to injury in the axolotl brain. The axolotl is a champion of regeneration with the exceptional ability to regrow limbs, heart, spinal cord, tail and large portions of brain tissue. Axolotl brain regeneration thus provides us with an excellent model for examining the relationship between spontaneous and injury-induced NPC behavior and its relationship to olfactory stimuli. Our detailed analysis builds upon previous work that detected dividing NPCs in the axolotl *Ambystoma mexicanum* CNS and showed that the number of dividing NPCs declines with age [30]. We found proliferating NPCs in the VZs of the telencephalon, mesencephalon and rhombencephalon with activity that was variable along the rostrocaudal and dorsoventral neuraxes between these regions. In the VZ, dividing cells exhibited radial glial-like glial fibrillary acidic protein (GFAP)-positive phenotypes. Cells positive for the cell synthesis marker bromodeoxyuridine (BrdU) migrated laterally, dorsally and ventrally from the VZs and differentiated into either almost mature neurons positive for doublecortin (DCX) and/or neuronal nuclei (NeuN) or mature NeuN^+^ neurons by 2 weeks. There appears to be a relative reduction in the neuronal population, suggesting a degree of selective cell death. Large-scale telencephalon tissue removal stimulated VZ proliferation and tissue regeneration. We only detected this injury-induced proliferative and regenerative response in animals with an intact olfactory nerve, suggesting that an olfactory cue could stimulate the response. We present this data as the beginning of a thorough cellular and molecular analysis of the phenomenon of brain regeneration in axolotls and its relation to proliferation of stem cells within the CNS.

## Results

### Proliferation zones in the axolotl brain

**Figure 1:**
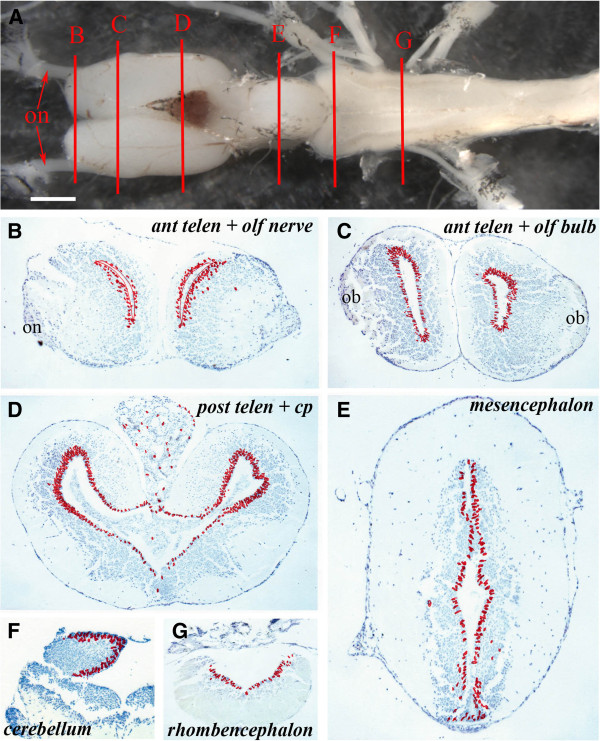
**Ventricular zones with variable proliferative activity can be detected throughout the axolotl central nervous system. **(**A**) Structure of the axolotl brain showing the levels at which sections ventricular zone proliferation was analyzed. **B** = anterior telencephalon and olfactory nerve, **C** = anterior telencephalon and olfactory bulb, **D** = posterior telencephalon, **E** = mesencephalon, **F** = cerebellum, **G** = rhombencephalon; on: olfactory nerve. Scale bar = 1 mm. (**B** –** G**) Cumulative BrdU^+^ cell numbers on five sections from each level depicted in **A** on which individual labeled cells are marked with a red dot. Regions of the brain are marked.; on: olfactory nerve; ob: olfactory bulb; cp: choroid plexus.

To locate proliferating neural progenitor cells and their progeny, juvenile axolotls (4 to 5 inches long; 3 to 4 months of age) were used. These animals do not metamorphose, become sexually mature in the larval form and retain exceptional regenerative ability throughout their lives. They were placed overnight (18 h) in water containing the cell synthesis marker BrdU. The brains were fixed, serially sectioned and BrdU^+^ nuclei identified on immunohistochemistry. The following regions were examined in transverse sections: anterior telencephalon and olfactory nerve (Figure 1A, level B), anterior telencephalon and olfactory bulb (Figure 1A, level C), posterior telencephalon and choroid plexus (Figure 1A, level D), mesencephalon (Figure 1A, level E), anterior rhombencephalon and cerebellum (Figure 1A, level F), and the mid rhombencephalon (Figure 1A, level G). To provide comparative data, the location of every BrdU^+^ cell in five adjacent sections was recorded at each level and represented as a summary section in the figures.

The axolotl brain is typified by the presence of a narrow, one- to three-cell layered VZ (matrix zone) adjacent to the ventricle. The VZ is surrounded by a wide region of uniformly spherical neurons surrounded by an axonal layer of varying thickness (Figure 1B-F). In axolotls sacrificed the morning after overnight BrdU incorporation, more than 99% of the BrdU-labeled cells were located in the VZ. Only occasional labeled cells with a neuronal phenotype were detected within the grey matter, or with a meningeal phenotype at the periphery or within the choroid plexus. At the level of the anterior telencephalon and olfactory nerve, BrdU^+^ cells were distributed uniformly in the dorsoventral axis of the VZ and no labeled cells were detected in the olfactory nerve (Figure 1B). At the olfactory bulb level (Figure 1C), BrdU^+^ cells were restricted to the VZ, but were apparently more abundant in dorsal and ventral regions than in the central VZ. Many BrdU^+^ cells were detected in the VZ of the posterior telencephalon (Figure 1D), with far more in the dorsal versus ventral VZ and more in the lateral than the medial VZ. Examination of cumulative cells labeled in the mesencephalon (Figure 1E) showed that considerably fewer BrdU^+^ cells were present in this region of the brain than in the dorsal telencephalon and that the number of labeled cells was consistent between the dorsal and ventral mesencephalic VZ. In the anterior rhombencephalon, at the level of the cerebellum, labeled cells were present both in the VZ and in the underlying parenchyma (Figure 1F) and in the mid-rhombencephalon (Figure 1G) there was a fairly uniform, but lower number of BrdU^+^cells along the VZ.

**Figure 2:**
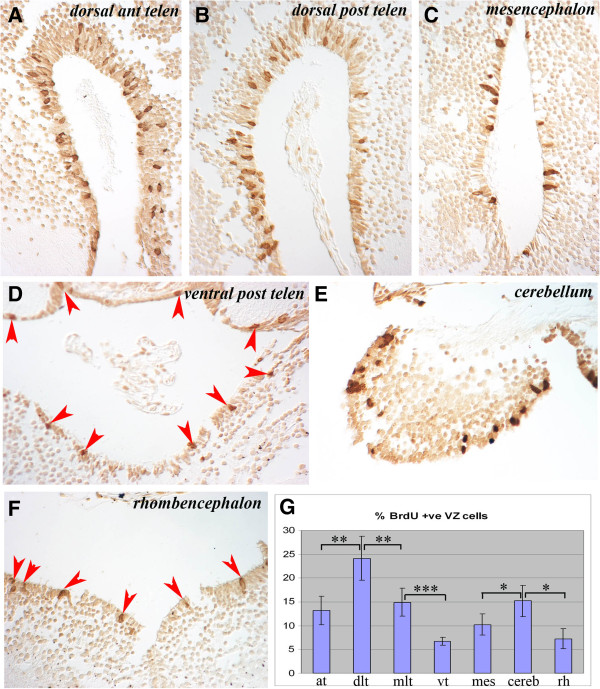
**Cells labeled with BrdU after an overnight incubation are largely retained in the ventricular zone throughout the neuraxis, which shows regional variation in proliferative activity. **(**A**-**F**) Examples of BrdU^+^ cells in the VZ of different brain regions show variation in numbers of labeled cells. Regions of the brain are marked. In **D** and **F**, BrdU^+^ cells are marked with red arrowheads. (**G**) Counts of proliferating VZ cells in different regions of the brain expressed as a percentage of total VZ cells. at: anterior telencephalon; BrdU: bromodeoxyuridine; cereb: cerebellum; dlt: dorsolateral telencephalon; mes: mesencephalon; mlt: mediolateral telencephalon; rh: rhombencephalon; vt: ventral telencephalon; VZ: ventricular zone. * significant (*P* = 0.025), ** very significant (*P* = 0.006), *** extremely significant (*P* = 0.0004) by Student’s *T*-test.

Figure 2 shows individual sections of the brain regions to highlight these differences in the abundance of BrdU^+^ VZ cells. The percentage of BrdU^+^ VZ cells in the different regions of the brain are show in Figure 2G. Figure 2A,B revealed similar proliferation levels in the dorsal region of the anterior versus the posterior telencephalon. But within this region, especially notable in Figure 2B, the lateral side of the VZ (left side of Figure 2B) had more proliferating cells than the medial side (right side of Figure 2B). This impression was confirmed by cell counts, where the dorsolateral posterior telencephalon had almost twice as many proliferating cells as the dorsomedial posterior telencephalon (24.2% versus 14.9%; Figure 2G). The ventral posterior telencephalon (Figure 2D) had low levels of proliferation (6.7%; Figure 2G), demonstrating how variable proliferation is within this one region of the brain. The mesencephalon (Figure 2C) had similar levels of proliferation to the anterior telencephalon (13.2% versus 10.2%, non-significant; Figure 2G). The cerebellum (Figure 2E) had far higher proliferative counts than its adjacent rhombencephalon (Figure 2F) (15.2% versus 7.2%; Figure 2G) with the rhombencephalon revealing the lowest levels of proliferation along with the ventral posterior telencephalon.

These data show that NPCs proliferate throughout the uninjured axolotl brain, with the highest levels detected in the dorsolateral telencephalon followed by the mediolateral telencephalon and cerebellum. There was an obvious dorsoventral difference in VZ proliferation in the posterior telencephalon that disappeared in more caudal regions. Occasional labeled cells were detected in parenchymal regions of animals sacrificed immediately after overnight BrdU labeling. The spherical morphology of these cells suggests that they represent either a small population of the migrating neuroblast progeny of NPCs that had completed their synthesis phase at the beginning of the labeling period or a small population of neurons undergoing DNA repair. The former hypothesis would suggest that neuroblasts migrate fairly quickly after NPCs complete division.

### Migration of labeled cells

To characterize the migration pattern of the VZ progeny into the surrounding differentiated neuronal layer, animals were placed overnight (18 h) in water containing BrdU and then kept in fresh water for 1, 2, 3 or 4 weeks before being sacrificed.

**Figure 3:**
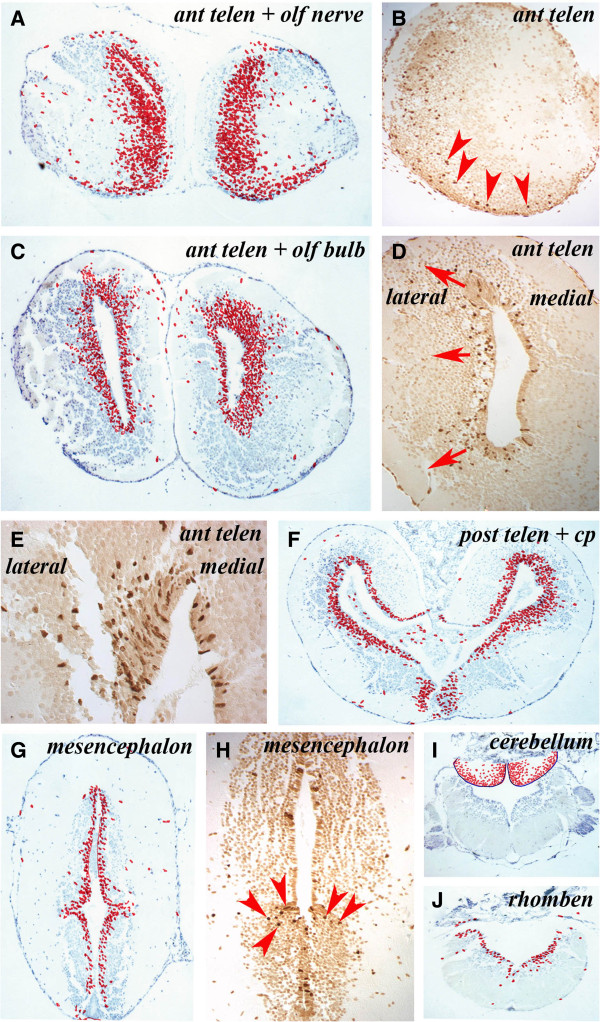
**Sections taken one week after overnight BrdU labeling show the migration of BrdU^+^ cells from the ventricular zone into the mantle zone at different rates at different levels of the central nervous system. **(**A**, **C**, **F**, **G**, **I**, **J**) Positions of labeled cells on five cumulative sections at the levels marked on the figure. The migration of BrdU^+^ cells outwards at different rates can be appreciated by comparing **A** and **C** with **G**. (**B**, **D**, **E**, **H**) Labeled cells on individual sections.

In animals sacrificed 1 week after BrdU labeling, 1-week-old BrdU^+^ cells had migrated out of the VZ into the adjacent neuronal layer, most prominently in the telencephalon followed by the cerebellum (Figure 3A,I). At the level of the anterior telencephalon and olfactory nerve, there was an expanded VZ zone of labeled cells and apparent streams of labeled cells migrating laterally and ventrally toward the olfactory bulb and nerve (Figure 3A) that could be readily detected in a single section (Figure 3B). In the anterior telencephalon and olfactory bulb, the expanded zone of labeled cells was more prominent on the lateral side of the VZ than the medial side (Figure 3C,D). This difference can be clearly seen in single sections such as the highlight of the dorsal part of the telencephalic VZ (Figure 3E). In the posterior telencephalon there was also a lateral expansion of the zone of labeled cells that was more prominent dorsally than ventrally (Figure 3F). In the mesencephalon, few labeled cells appeared to have migrated away from the VZ (Figure 3G) except at the junction between the thalamus and the hypothalamus where labeled cells spreading laterally could be observed (Figure 3H, red arrowheads). There was a high degree of movement among BrdU^+^ cells from the VZ to the parenchyma of the cerebellum (Figure 3I). Similar to the mesencephalon, very few BrdU^+^ cells had migrated from the VZ into the parenchyma of the rhombencephalon (Figure 3J). In this and other sections, some BrdU^+^ cells could be seen far from the VZ, sometimes at the edge of the brain. We have not studied these cells in detail but suggest they are proliferating *in situ*; for example BrdU^+^ meningeal cells could occasionally be observed. These zones of higher migration rates correlated with the zones of high proliferation (Figure 2G).

**Figure 4:**
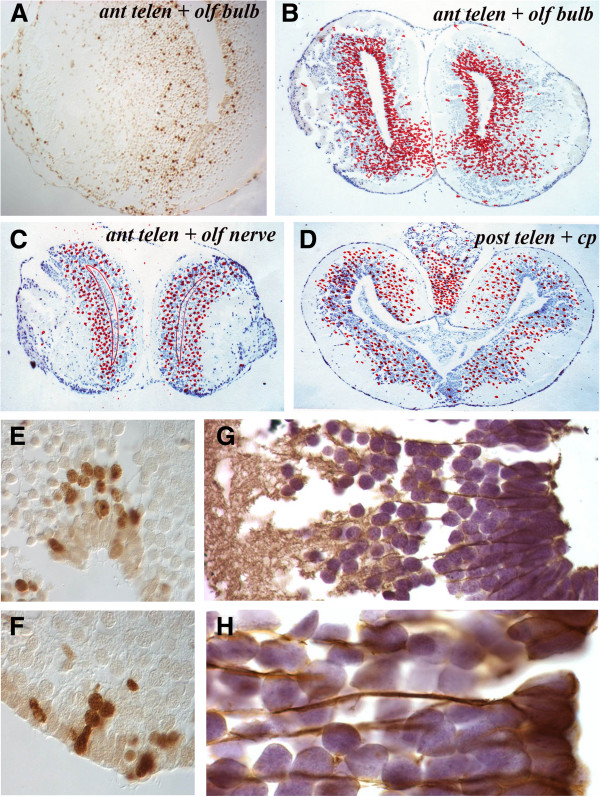
**Migration patterns and glial fibrillary acidic protein expression in the telencephalon. **(**A**) Single section through the anterior telencephalon and olfactory bulb taken 4 weeks after overnight BrdU labeling shows a stream of cells moving laterally into the olfactory bulb. (**B**) Cumulative positions of BrdU^+^ cells in the anterior telencephalon and olfactory bulb level 4 weeks after overnight labeling. (**C**) Cumulative positions of BrdU^+^ cells in the anterior telencephalon and olfactory nerve level 4 weeks after overnight labeling showing a decreased number of BrdU^+^ cells in the VZ. (**D**) Cumulative positions of BrdU^+^ cells in the posterior telencephalon 4 weeks after overnight labeling showing a decreased number of BrdU^+^ cells in the VZ. (**E**,**F**) Patterns of BrdU^+^ cells in and adjacent to the VZ 3 weeks after overnight labeling. (**G**,**H**) Glial fibrillary acidic protein immunocytochemistry of the normal telencephalon at low power (**G**) and high power (**H**) to show the cytoplasmic extensions of radial glial cells. BrdU: bromodeoxyuridine; VZ: ventricular zone.

Over the ensuing weeks the patterns of movement within the parenchyma appeared to continue, with BrdU^+^ cells moving more laterally and ventrally away from the VZs of the telencephalon and cerebellum and, to a lesser degree, in the less active mesencephalon and rhombencephalon. At the anterior telencephalon and olfactory bulb level (Figure 4A,B), for example, there was a widespread ventral migration both towards the olfactory bulb and medially whereas, more anteriorly at the level of the olfactory nerve, there was a much reduced and more generalized migration of cells (Figure 4C). This was the most common response, also apparent at the posterior telencephalon level (Figure 4D) where BrdU^+^ cells were distributed throughout the neuronal layer with a reduced number of labeled cells remaining in the VZ.

In less active regions, intensely labeled BrdU^+^ cells were more frequently observed within the VZ at later time periods. By contrast, in more active regions, paler stained nuclei were seen within the VZ, suggesting these were the progeny of cell divisions. Within the parenchyma, BrdU^+^ cells were often seen closely adjacent to labeled VZ cells as clumps (Figure 4E) or as lines (Figure 4F), suggesting lineage relationships.

### Phenotype of labeled cells

**Figure 5:**
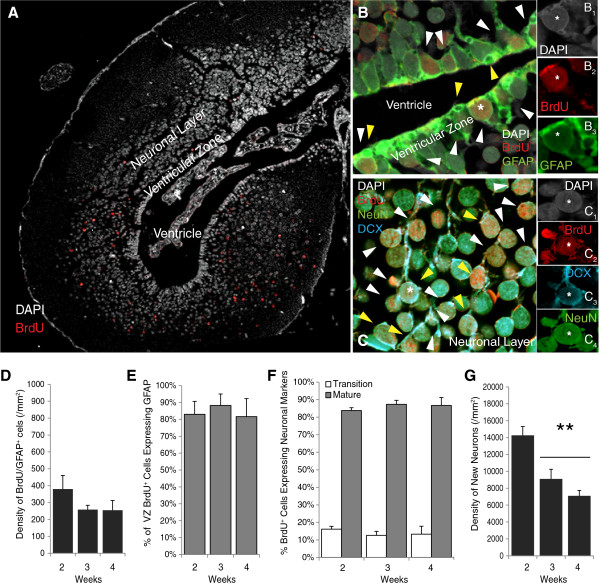
**Two weeks after BrdU labeling, new cells have migrated and differentiated into neurons and a few new cells are retained in the ventricular zone and express GFAP. **(**A**) Confocal image at 10× magnification shows that, in the uninjured axolotl telencephalon, most BrdU^+^ cells have migrated into the mantle zone 2 weeks after labeling. A few BrdU^+^ cells are retained in the VZ. (**B**,**C**) Confocal images taken at 40× objective (2.3× digital zoom) of sections stained with 4^′^-6-diamidino-2-phenylindole (in white; B_1_ and C_1_) and antibodies against (**B**) BrdU (in red; B_2_) and GFAP (in green; B_3_) or (**C**) BrdU (in red; C_2_), DCX (in blue; C_3_), NeuN (in green; C_3_). The image in (**B**) shows that very few BrdU^+^ cells are retained in the VZ by 2 weeks after overnight BrdU labeling, but those that do remain and express GFAP may represent new radial glia-like cells. (**C**) by 2 weeks, all BrdU^+^ cells in the mantle zone express the mature neuronal marker NeuN (white and yellow arrows) and a small percentage of the NeuN^+^ neurons retain DCX expression (yellow arrows) and are likely transitioning into mature neurons. (**D**) The relatively small densities (versus **F**) of BrdU/GFAP^+^ cells retained in the VZ are resilient across weeks 2 to 4 after BrdU labeling. (**E**) Most BrdU^+^ VZ cells express GFAP. (**F**) All BrdU^+^ cells in the neuronal layer express NeuN and a few NeuN^+^ neurons retain DCX expression. These percentages are consistent across weeks 2 to 4 after BrdU labeling. (**G**) New neurons are either vulnerable to cell death or are still migrating because their densities significantly decrease between weeks 2 and 3 (**P <0.001). BrdU: bromodeoxyuridine; DCX: doublecortin; GFAP: glial fibrillary acidic protein; VZ: ventricular zone.

Most VZ cells contain flat apical surfaces lining the ventricle and pointed basal surfaces with elongated cytoplasmic extensions extending to the pial surface of the brain. These morphologies are consistent with the classical radial glial cell morphologies of lower vertebrates [41-46] and the VZ cells all stain with GFAP (Figure 4G,H). Their glial fibers form columns with spherical NeuN^+^ neuronal cells arranged in columns between the radial glial fibers (Figures 4H and 5C). A minority of GFAP^+^ VZ cells had smaller nuclei and appeared not to stain with GFAP (Figure 5B, yellow arrows).

To examine the differentiative properties of proliferative VZ cells, sections were immunolabeled to detect the glial markers GFAP (astrocytes) and NG2 (early oligodendroctyes) or the neuronal markers DCX (immature neurons) and/or NeuN (mature neurons) expressed by BrdU^+^ cells (Figure 5A-C). The densities of BrdU^+^ cells located in the VZ (Figure 5D) and neuronal layers (Figure 5G) and their phenotypes (Figure 5B,C,E,G) in uninjured animals that were sacrificed 2, 3 and 4 weeks after BrdU labeling were then quantified under confocal microscopy. We have not been able to detect NG2^+^ cells with commercially available antibodies, previously and in the current study, which could reflect that these antibodies were raised against a non-homologous region of the mammalian protein or that NG2^+^ oligodendrocyte precursor cells are seldom found in the uninjured axolotl CNS.

In the telencephalon, the migration of BrdU^+^ cells out of the VZ appeared complete by 2 weeks because the total percentage of BrdU^+^ cells that were retained in the VZ (2.50 ±0.53%, 2.0 ±0.1% and 1.1 ±0.3%, respectively; *F*(2,10) = 1.14; *P* = 0.36.) versus those that had migrated into the parenchymal neuronal layer (97.50 ±0.53%; 98.0 ±0.4% and 98.9 ±3.6%, respectively; (*F*(2,10) = 1.14; *P* = 0.36) remained consistent across weeks 2 to 4. Figure 5D shows that the density of BrdU^+^ cells retained in the VZ is resilient, because although the densities are relatively small, they did not change across weeks 2 to 4 after labeling (*F*(2,10) = 1.31; *P* = 0.31). The majority (84.0 ±4.5%) of these BrdU^+^ VZ cells express GFAP (*F*(2,10) = 1.56; *P* = 0.86; Figure 5E), suggesting that some progeny either retain a radial-glial like progenitor cell phenotype or differentiate into ventricular zone astrocytes. If these cells do represent a population of new radial glia-like progenitors, their retention of BrdU suggests that they either remain quiescent or do not undergo multiple divisions within the VZ. These speculations could be tested in future experiments. All spherical BrdU^+^ cells that had migrated out of the VZ into the surrounding neuronal layer expressed the mature neuronal protein NeuN. A small fraction (approximately 14.2 ±1.6%) of these BrdU/NeuN^+^ cells retained expression of the immature neuronal marker DCX, suggesting that they were transitioning to a mature state (Figure 5F). These data suggest that new neurons mature along similar timelines in the uninjured axolotl telencephalon and rodent hippocampus [47]. In contrast to the BrdU/GFAP^+^ cells retained in the VZ, BrdU^+^ neurons (DCX and/or NeuN^+^) that had migrated into the parenchyma appeared to either be vulnerable to cell death or continue to migrate because their densities decreased across weeks 2 to 4 (*F*(2,10) = 14.66; *P* = 0.001). Specifically, relative to 2-week-old neurons, fewer 3-week-old (*P* = 0.005) and 4-week-old (*P* = 0.001) neurons were detected (Figure 5G). If this phenomenon reflects the attrition that has been well documented in the rodent hippocampus [47] then it may be occurring somewhat more latently in the axolotl CNS, although we have not investigated other possibilities and we did not directly measure cell death in either the VZ or parenchyma.

### Characteristics of telencephalic regeneration

To begin an analysis of the relationship between VZ proliferation in response to damage, the olfactory nerve and regenerative ability we have investigated various regenerative regimes in the telencephalon. We have replicated the striking findings that the olfactory system plays a role in regeneration that were described over 50 years ago by Kirsche [9,34-36] and others [37,38]. Although both telencephalons can, remarkably, regenerate in their entirety after simultaneous removal [48], we have found that removing tissue segments from one telencephalon while leaving the other intact as a control or removing tissue segments as well as severing the olfactory nerve provides better insights into the underlying mechanisms of regeneration.

**Figure 6:**
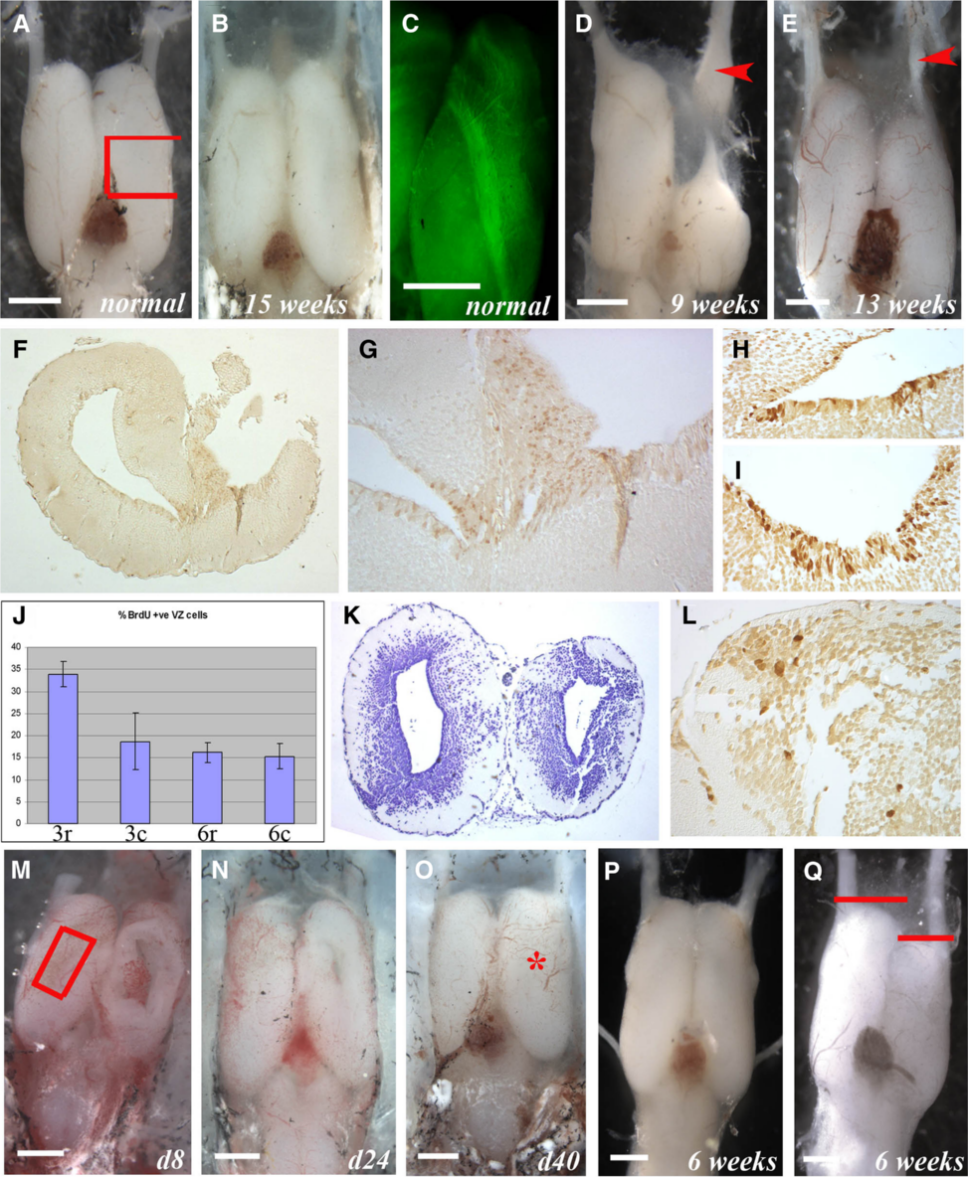
**Regeneration of the axolotl telencephalon. **(**A**) Normal forebrain (ablated region marked with red box). (**B**) 15 weeks later showing complete regeneration. (**C**) RT97 wholemount showing the medial olfactory tract. (**D**) 9 weeks after removal of the right anterior third and olfactory bulb showing failure of regeneration and swelling of the regenerating olfactory nerve (arrowhead). (**E**) After removal of the olfactory bulb (right) the telencephalon heals but does not regenerate until the olfactory nerve regenerates (arrowhead = olfactory nerve swelling). (**F**-**L**) Regeneration after removal of the middle third of the telencephalon (as in **A**). After 3 weeks (**F**, **G**) there is no regeneration but an increase in BrdU^+^ cells in the damaged VZ (right). Close-up of the undamaged (**H**) and damaged (**I**) VZ shows more BrdU^+^ cells in **I**. **J**, BrdU^+^ counts in the VZ from 3-week regenerating (3r), 3-week undamaged (3c), 6-week regenerating (6r), 6-week undamaged (6c) with standard deviations. K, after 6 weeks there is restoration of the damaged side (right) but not full tissue replacement. After overnight BrdU labeling at 6-weeks there is local proliferation at the cut site (**L**). (**M**-**Q**) Regeneration after dorsal pallium removal (red box in **M**). **M**, damage site (right) after 8 days. **N**, after 24 days there is near complete regeneration. **O**, 40 days after removal the left and right telencephalons are indistinguishable (damage site marked with red star). **P**, regenerated brain 6 weeks after removal of the right dorsal pallium (as in **M**) shows complete regeneration. **Q**, 6 weeks after right dorsal pallium removal and olfactory nerve severing there is wound repair but shrinkage of the right telencephalon while the olfactory nerve regenerates. Red bars show the left/right difference in telencephalon length. Scale bars = 1 mm.

Removed telencephalon segments, such as the middle third segment, regenerated within 12 to 15 weeks in 4- to 5-inch-long animals (compare right telencephalon in Figure 6A with Figure 6B). Regenerated segments are externally indistinguishable from undamaged segments at this time frame although, histologically, the full thickness of the telencephalon has not always recovered. Although the tissue removals described here sever the median olfactory tract (Figure 6C), we have not tested these animals to determine whether behavioral function has returned nor have we performed electrophysiological assessment of connectivity. We have only assessed the regeneration of the damaged site morphologically. Overnight BrdU labeling experiments during the process of regeneration revealed that NPC proliferation in the VZ of the damaged telencephalon, which often exhibited a ventral thickening (Figure 6F,G), is up-regulated relative to NPC proliferation in the VZ of the undamaged contralateral telencephalon by approximately 3 weeks after the damage (compare Figure 6H and I, counts in J). After 6 weeks, the damaged area had healed enough to restore the appearance of a well-defined ventricle (Figure 6K) and NPC proliferation in the VZ of the damaged telencephalon had subsided to resemble that in the VZ of the control telencephalon (Figure 6J). However, at this later stage, dividing NPCs were additionally detected in the cells surrounding the wound margins immediately after a BrdU pulse (Figure 6L). By contrast, BrdU^+^ cells are never detected in the undamaged parenchyma immediately after a short BrdU exposure. These data suggest that injury-induced regeneration of the telencephalon may be accomplished initially by a cue that stimulates the proliferation of NPCs in the VZ and then latently by a cue that stimulates a more local proliferative response. One possibility is that the first response may recruit a large number of cells to replace missing tissue whereas the second response may harmonize the cut surfaces. These conjectures are based on the assumption that BrdU uptake indicates cell cycling but other explanations are equally valid, such as that the localized BrdU^+^ cells in Figure 6J could be simply repairing their DNA.

We found several lines of evidence consistent with the hypothesis that telencephalic regeneration may be stimulated by a cue provided by the olfactory nerve. In cases where the olfactory nerve is severed along with removal of the anterior third of the telencephalon, regeneration only begins once the olfactory nerve regrows enough to contact the residual portion of the telencephalon. During this period the remaining part of the telencephalon has undergone wound healing but not regeneration, and regrowth is suspended until contact is made. For example, by approximately 9 weeks after anterior telencephalon damage, which is coincident with the time frame that a removed middle third segment of the telencephalon would have nearly completed regeneration (for example, Figure 6B), the re-growing olfactory nerve exhibits a distal swelling as contact is made (Figure 6D, red arrowhead). This swelling was described as containing olfactory neuroblasts [9,34-38]. The same phenomena of wound healing, delayed regeneration and olfactory nerve swelling can also be seen in more precise tissue removal, for example of only the olfactory bulb (Figure 6E, red arrowhead).

A less invasive procedure that does not remove the olfactory bulb and where the effect of the olfactory nerve can readily be seen is after the removal of a rectangular segment of the dorsal pallium (Figure 6M). In one group of animals where the dorsal pallium was removed without damage to the olfactory nerve or bulb, the damage heals and the missing tissue is rapidly replaced. After 24 days the wound size has vastly reduced (Figure 6N) and by 40 days looks to have completely regenerated (Figure 6O). Sections reveal, however, that the full thickness of the dorsal pallium has not yet regenerated by this time after damage. In the second group of animals in which the olfactory nerve is also severed at the time of dorsal pallium removal, the damage heals successfully, but the telencephalon shrinks in length and does not begin to regenerate its size until the olfactory nerve returns. This can be seen by comparing Figure 6P, taken 6 weeks after dorsal pallium removal on the right side showing good regeneration, with Figure 6Q, taken at the same time after dorsal pallium removal plus olfactory nerve severing. The latter shows repair of the wound site but no regeneration at this time as there is a clear reduction in size of the right telencephalon. While Kirsche [9,34-36] and others [37,38] suggested that the olfactory nerve may be a source for cells that regenerate the telencephalon, data from the current study and work in mammals, teleosts and reptiles suggests that the more likely explanation is that the olfactory nerve provides a trophic stimulus to VZ and perhaps parenchymal NPCs. Future experiments will attempt to determine the nature of this trophic regenerative stimulus.

## Discussion

The regenerative ability of the axolotl is unparalleled among vertebrates and includes the ability to regenerate large parts of the brain. By identifying the mechanisms that control this process in the axolotl we hope to gain insight into why this process does not occur spontaneously in higher vertebrates, including man, despite the presence of forms of CNS plasticity and NPCs capable of proliferating and generating neurons that incorporate into functional neural circuits [1,4-8]. Brain regeneration, at least in response to incisional or stab wounds, occurs in fish, amphibians and reptiles throughout life [10,13,21,23,26]. Although NPCs are distributed throughout the neuraxis of adult mammals [1,5,8], neurons are only spontaneously generated in highly restricted areas [3,4] and only extremely limited abortive neurogenesis occurs in response to injury in extra-neurogenic regions (for review see [49]). Here we characterize the effects of removing variably-sized telencephalon sections on NPC proliferation and new cell migration in the axolotl to establish a model with which to investigate the mechanisms of brain regeneration.

The axolotl VZ, like other Urodeles, retains embryonic characteristics that include the presence of abundant GFAP^+^ radial glia with nuclei that line the ventricle and long cytoplasmic processes that extend to the pial surface [41-46]. New cells appear to migrate away from the VZ along these extensions to terminally differentiate into neurons once they reach the mantle zone. This process appears to be complete within 2 weeks, at which point all cells that have entered the mantle zone express the mature neuronal marker NeuN. A few of these cells retain expression of the immature neuronal marker DCX even 4 weeks after their birth as they transition from an immature to mature status. Why a small population of new neurons exhibits delayed maturation is unclear. Similar to new mammalian neurons, these new axolotl neurons appear vulnerable to cell death because their densities decrease in the month after their birth. Whether these dying cells supply a stimulus to VZ NPCs to maintain spontaneous neuronal turnover would be interesting to test in future experiments. A small population of the new cells remains in the VZ and expresses GFAP. These cells likely replenish the VZ radial glia population as we did not detect any GFAP^+^ cells in the mantle zone, which is consistent with previous data collected in the axolotl [44,45], and in fish and reptiles [17,27].

Spontaneous NPC proliferation and neurogenesis occurred in the VZs throughout the axolotl brain but some zones, such as the dorsal and ventral telencephalon, exhibited elevated activity levels. Whether neuronal turnover is higher in these highly active regions is currently unclear. The axolotl does continue to grow in adulthood so these new neurons could also contribute to areas of elevated growth. Indeed, spontaneous VZ NPC proliferation varies across 16 zones in the adult zebrafish [15,16]. This situation in axolotls is likely related to their pedamorphic nature, as adult newts show VZ proliferation only in the telencephalon [31] as do reptiles [26]. Future work testing how widespread spontaneous and injury-induced NPC proliferation and neurogenesis is in the CNS of metamorphosed axolotls would be an interesting test of their regenerative potential. When studied over time, BrdU^+^ cells appeared to migrate from these regions of high activity in a lateral and dorsoventral rather than medial direction into the mantle zone. A ventral stream of BrdU^+^ cells originating from the ventromedial surface of the anterior telencephalon toward the olfactory bulbs resembled the rostral migratory stream in the mammalian brain and was consistent with reports in other vertebrate brains. Similar to differential rates of NPC proliferation throughout the brain, migration of new cells into the mantle varied across brain regions with the most active areas occurring in the telencephalon and the cerebellum with far less activity in the mesencephalon and rhombencephalon.

It is important to consider whether the ability to proliferate and generate new neurons *per se* endows the brain with regenerative capacity or whether regenerative capacity is a unique property of particular taxa [11]. Considering the former possibility, brain regeneration, at least in response to incisional or stab wounds, occurs in fish and reptiles that show extensive spontaneous NPC proliferation throughout the adult VZ. In birds and mammals, proliferation occurs in more restricted proliferative zones [21] and neurogenesis is restricted to regions such as the higher vocal center of birds [50] and the hippocampal dentate gyrus, olfactory epithelium and olfactory bulbs of mammals [51]. Considering the latter possibility, Urodele cells or their niches may be unique. Although spontaneous NPC proliferation is detected throughout the VZs of the axolotl brain, it is only detected in the VZ of the telencephalon in adult newts [31]. Yet 70% of the optic tectum [33] and the entire set of dopaminergic neurons in the ventral midbrain [31,32] can be regenerated in response to tissue removal or chemical injury in the adult newt. In these circumstances, injury induces normally quiescent NPCs to proliferate until the lost cells are regenerated. These data suggest that the ability to regenerate neurons is not simply a by-product of continual growth, and gives us a greater opportunity for investigating the nature of the mechanisms behind spontaneous and injury-induced NPC proliferation and neuronal regeneration. As a first step, we have confirmed the importance of the olfactory nerve as a source of the injury-induced stimulus for telencephalic regeneration in the axolotl. Discovering the nature of this stimulus will be the subject of our future investigations.

## Conclusion

There is a continual generation of neuronal cells from neural progenitor cells located within the ventricular zone of the axolotl brain. Variable rates of proliferation were detected across brain regions and variable migration routes observed over time. The telencephalon and the cerebellum were the most active brain regions and ventricular zone cells either generated more radial glial cells, which remained in the VZ, or new neurons. New neurons appeared to undergo a similar attrition to that seen in mammals as their numbers declined over time. These neural progenitor cells in the VZ appear to mediate complete telencephalic tissue regeneration through an injury-induced olfactory cue. Identification of this cue is our future goal.

## Methods

### Animals

Axolotls (*Ambystoma mexicanum*) of 4 to 6 inches in length were purchased from the Ambystoma Genetic Stock Centre (University of Kentucky, Lexington, KY, USA). They were maintained in 40% Holtfreter’s solution and treated in accordance with the University of Florida’s Institutional Animal Care and Use Committee’s regulations. For all the experiments, group sizes were between two and four animals for each data point.

### BrdU labeling and histology

Animals were placed in 1 L of Holtfreter’s solution containing 50 mg of the cell synthesis marker BrdU (Sigma Aldrich, St. Louis, MO, USA) overnight (18 hrs) and then either sacrificed or placed into fresh Holtfreter’s solution for another 1, 2, 3 or 4 weeks before being sacrificed. At each time point, the animals were anaesthetized in 1:5,000 tricaine methanesulfonate (MS222; Argent Chemical Laboratories, Redmond, Washington, USA) before their brains were dissected out of the cranium and post-fixed overnight in 4% paraformaldehyde at 4°C. The brains were dehydrated, embedded in paraffin wax and sectioned at 10 μm.

### Immunocytochemistry

To localize dividing NPCs in brains fixed after overnight exposure to BrdU and surviving new cells in brains fixed 1, 2, 3 or 4 weeks after BrdU exposure, BrdU^+^ cells were revealed enzymatically with 3,3’-diaminobenzidine tetrahydrochloride and imaged under light microscopy. Sections were dewaxed, incubated in 0.3% H_2_O_2_ in 0.1 M Tris-buffered saline (pH 7.4) for 10 min to quench endogenous peroxidase, and then in 2 M HCl for 20 min at 37°C to denature DNA. They were then blocked in normal donkey serum (Tris-buffered saline, 3% normal donkey serum and 0.1% triton-X) and incubated overnight at 4°C with rat anti-BrdU (OTB0030CX, 1:500; AbD Serotec, Raleigh, NC, USA). The next day, the sections were incubated in biotinylated secondary anti-rat IgG (Jackson ImmunoResearch, West Grove, PA; USA, 712-065-150, 1:500) for 4 h at room temperature and then in avidin-conjugated horseradish peroxidase (PK6100; Vector Laboratories, Burlingame, CA, USA) for 2 h before being reacted in a solution of 0.02% 3,3’-diaminobenzidine tetrahydrochloride (D9015; Sigma Aldrich) and 0.5% H_2_O_2_.

After BrdU immunocytochemistry, the numbers of positive cells were counted throughout the brain as the percentage positive cells in the VZ. To visualize their localization and their spread from the VZ over time, BrdU^+^ cells were also localized on photographs of the relevant sections by marking their position with a red dot. The position of every positive cell on five adjacent sections was marked on the photographs.

To identify the phenotype of surviving new cells, the expression of neuronal or glial proteins by BrdU^+^ cells was quantified using fluorophore-conjugated secondary antibodies. After overnight incubation in rat anti-BrdU, these sections were incubated for 4 h at room temperature in Cy3-conjugated anti-rat immunoglobulin G and then mouse anti-NeuN (1:500; Chemicon, Temecula, CA, USA) and goat anti-DCX (1:500; Santa Cruz Biotechnology, Santa Cruz, CA, USA) to detect neuronal phenotypes or rabbit anti-chondroitin sulfate proteoglycan (NG2; 1:500; Chemicon) and chicken anti-GFAP (EnCor Biotech, Gainesville, FL, USA) to detect glia and then the appropriate maximally cross-adsorbed cyanine 5- and fluorescein isothiocyanate-conjugated secondary antibodies (1:500; Jackson ImmunoResearch). Note that we did not detect NG2^+^ cells in the axolotl brain with the antibody used.

For whole-mount immunocytochemistry, brains were dissected from the animal and fixed overnight in 4% paraformaldehyde. They were washed several times in PBS-Tween (PBST) and then placed in RT97 antibody (1:50; Developmental Studies Hybridoma Bank, University of Iowa, Iowa City, IA, USA) made up in PBST at 4°C for 6 days. After washing for 1 day in PBST, immunoreactivity was visualized with a fluorescent donkey anti-mouse immunoglobulin G (1:200; Alexa Fluor, Life Technologies, Grand island, NY, USA) made up in PBST for 2 days, washed extensively and then cleared with 80% glycerol.

### New cell densities and phenotyping

New cell densities were confirmed in the neuronal mantle by counting the total number of BrdU^+^ cells in images of non-overlapping telencephalon sections. New cell densities in the VZ were confirmed by counting BrdU^+^ cells on VZ areas measured using Zeiss Zen software, because the VZ only comprised a portion of an imaged area. We examined more than 100 BrdU^+^ cells on triple fluorescent-stained sections (two to four sections per axolotl) for the co-expression of neuronal or glial proteins using a Zeiss meta LSM 710 fully spectral laser scanning confocal microscope (with 405, 488, 510, 543 and 633 laser lines, Carl Zeiss, Jena, Germany). BrdU^+^ cells were considered co-labeled when a full ‘z-dimension’ scan revealed its BrdU/4^′^-6-diamidino-2-phenylindole^+^ nucleus was unambiguously associated with a lineage marker such as the immature neuronal protein DCX [52-54], the mature neuronal protein NeuN [55], the oligodendrocyte precursor protein NG2 [56] or the astrocyte protein GFAP [57]. The percentage of BrdU^+^ cells expressing each phenotype was calculated.

### Brain regeneration experiments

Operations were performed by anaesthetizing animals in 1:5,000 MS222. The skin was removed from above the cranium on three sides and flapped back caudally to expose the cartilaginous cranium roof. This was also cut on three sides and bent back caudally to reveal the telencephalon and mesencephalon. Various operations were performed on one telencephalon using fine iridectomy scissors. These operations included, first, removal of the middle third of the telencephalon while leaving the olfactory bulb and olfactory nerve intact; second, removal of the anterior third including severing the olfactory nerve and removal of the olfactory bulb; and third, removal of a rectangular piece of the dorsal pallium with or without severing the olfactory nerve. All operations were carefully performed to prevent damage to the choroid plexus, and to ensure minimal blood loss. Following the relevant operation, the cartilage flap of the cranium was replaced followed by the skin. The skin flap was kept in place with superglue. The animals were then replaced in Holtfreter’s solution. All operated animals survived the surgery and showed apparently normal behavior in the ensuing weeks. At weekly intervals, operated brains were sampled for histology and cell proliferation after overnight BrdU incorporation. Nissl staining was performed for structural analysis and BrdU immunocytochemistry performed as described above.

### Statistical analyses

Analyses were performed using STATISTICA software (StatSoft; Tulsa, OK, USA). Analyses of variance were used to explore the effects of the independent variables of survival time on measures of neurogenesis (new cell densities and new cell phenotypes). Newman Keuls post hoc tests were used to reveal group differences. All data is represented in figures as the group average ± standard error of the mean and significance for all statistical analyses was set at α = 0.05.

## Abbreviations

BrdU: 5-bromo-2’-deoxyuridine; CNS: central nervous system; DCX: doublecortin; GFAP: glial fibrillary acidic protein; NeuN: neuronal nuclei; NG2: chondroitin sulfate proteoglycan; NPC: neural progenitor cells; PBST: phosphate-buffered saline-Tween; VZ: ventricular zone.

## Competing interests

The authors declare that they have no competing interests.

## Authors’ contributions

MM designed and performed the experimental analyses and wrote the manuscript; LM performed the immunohistochemical analyses, confocal microscopy and cell counting; BO designed the experiments, analyzed the data and co-wrote the manuscript. All authors read and approved the final manuscript.
